# Oxypurinol protects renal ischemia/reperfusion injury *via* heme oxygenase-1 induction

**DOI:** 10.3389/fmed.2023.1030577

**Published:** 2023-03-09

**Authors:** Hye Bin Kang, Chae Kyu Lim, Jongwan Kim, Sang Jun Han

**Affiliations:** ^1^Department of Biotechnology, College of Fisheries Sciences, Pukyong National University, Busan, Republic of Korea; ^2^Department of St. Mary Pathology and Laboratory Medicine, Busan, Republic of Korea; ^3^Department of Medical Laboratory Science, Dong-eui Institute of Technology, Busan, Republic of Korea

**Keywords:** oxypurinol, acute kidney injury, renal ischemia/reperfusion, oxidative damage, apoptosis

## Abstract

Renal ischemia/reperfusion (I/R) injury is a major cause of acute kidney injury (AKI) by increasing oxidative stress, inflammatory responses, and tubular cell death. Oxypurinol, an active metabolite of allopurinol, is a potent anti-inflammatory and antioxidant agent. To investigate the therapeutic potential and underlying mechanism of oxypurinol in ischemic AKI, C57BL/6 male mice were intraperitoneally injected with oxypurinol and subjected to renal I/R or sham surgery. We found that oxypurinol-treated mice had lower plasma creatinine and blood urea nitrogen levels and tubular damage (hematoxylin-and-eosin staining) compared to vehicle-treated mice after renal I/R injury. Furthermore, oxypurinol treatment reduced kidney inflammation (i.e., neutrophil infiltration and MIP-2 mRNA induction), oxidative stress (i.e., 4-HNE, heme oxygenase-1 [HO-1], 8-OHdG expression, and Catalase mRNA induction), and apoptosis (i.e., TUNEL or cleaved caspase-3-positive renal tubular cells), compared to vehicle-treated mice. Mechanistically, oxypurinol induced protein expressions of HO-1, which is a critical cytoprotective enzyme during ischemic AKI, and oxypurinol-mediated protection against ischemic AKI was completely eliminated by pretreatment with tin protoporphyrin IX, an HO-1 inhibitor. In conclusion, oxypurinol protects against renal I/R injury by reducing oxidative stress, inflammation, and apoptosis *via* HO-1 induction, suggesting its preventive potential in ischemic AKI.

## Introduction

Acute kidney injury (AKI) is significantly associated with morbidity and mortality owing to serious complications, such as electrolyte imbalance, gastrointestinal bleeding, and hospital-associated infections (1). Renal ischemia/reperfusion (I/R) injury, defined as the restriction of blood supply to the kidney (ischemia) followed by blood flow restoration and reoxygenation (reperfusion), is a major cause of perioperative AKI (2). Tubular cell death is caused by a combination of renal tubular necrosis, which occurs because of significant energy loss in renal tubular cells during ischemia, and apoptosis, which is activated during reperfusion (3). Additionally, chemokines and cytokines released from renal cells and leukocytes induce a strong inflammatory response during the reperfusion phase, attracting the infiltration of leukocytes such as neutrophils to cause additional renal tubular injury (4). However, the mechanisms of AKI are complex, and many of these pathways remain unknown.

Renal I/R injury is associated with the generation of reactive oxygen species (ROS) that exceed defensive antioxidant systems and consequent oxidative damage to macromolecules, such as proteins, DNA, and lipids (2). Nuclear factor erythroid 2-related factor 2 (Nrf2) modulates several cellular antioxidant mechanisms that limit oxidative stress during I/R-induced kidney injury. Under normal conditions, Nrf2 interacts with the negative regulator Kelch-like ECH-associated protein 1 (Keap1), and is degraded by ubiquitination. When activated, Nrf2-bound Keap1 is inactivated, and Nrf2 proteins freely translocate into the nucleus and bind to antioxidant response elements (ARE) encoding antioxidant and detoxifying enzyme genes, including heme oxygenase-1 (HO-1) (5–7). Among Nrf2 regulated genes, HO-1 has received significant attention in treating numerous kidney diseases, owing to its crucial cytoprotective role in various pathophysiological conditions, including I/R injury-, LPS-, and nephrotoxin-induced renal injury (8).

Oxypurinol is a well-known primary metabolite of allopurinol that is specifically used to prevent gout, specific types of kidney stones, and hyperuricemia (9). Previous studies have shown that pretreatment of allopurinol attenuated renal I/R injury by anti-oxidative (10), and anti-inflammatory (11) effects. In addition, Zhou et al. demonstrated that pretreatment of allopurinol prevents renal I/R injury by inhibiting high mobility group box 1 (HMGB1) which is a novel marker of inflammation (12) expression in a rat model. Compared to allopurinol, oxypurinol reportedly has biological properties, including anti-oxidative (13, 14), anti-inflammatory (13), and anti-cell death (15) activities, in diverse pathological conditions. For examples, Escobar et al. (13) reported that oxypurinol treatment protected against oxidative damage and upregulated pro-inflammatory genes in acute pancreatitis. LoBalsamo et al. (16) also demonstrated that oxypurinol protects the heart from I/R injury in rats. One clinical study reported that a 6-month oxypurinol therapy reduced mortality in patients with both high serum urate and chronic heart failure (17). However, the effect and underlying pathogenic mechanisms of oxypurinol on I/R-induced AKI remain to be elucidated. In this study, we evaluated the therapeutic potential of oxypurinol for ischemic AKI and investigated its underlying mechanism.

## Materials and methods

### Animal preparation

All animal surgeries were approved by the Institutional Animal Care and Use Committee (IACUC) of Pukyong National University and conducted in accordance with the Guide for the Care and Use of Laboratory Animals published by the US National Institutes of Health (NIH Publication No. 85–23, revised 2011). Eight-week-old C57BL/6 male mice (20–25 g) were anesthetized intraperitoneally with pentobarbital sodium (50 mg/kg; Hanlim Pharma Co., Seoul, Korea) and subjected to left nephrectomy and 30-min right renal ischemic periods to clearly see the protective effect of oxypurinol on ischemic AKI (18–20). The sham-operated mice underwent the same surgical procedure without renal ischemia (21). Some mice were intraperitoneally injected with oxypurinol (25 or 50 mg/kg, Sigma-Aldrich) or vehicle (DMSO, 2.5 mL/kg, Sigma-Aldrich) at 24 and 1 h before surgery or with hemin (25 mg/kg, Sigma-Aldrich) or vehicle (12.5 mM, pH 7.3, NaOH, 10 mL/kg) 24 h before surgery based on previous studies (22, 23). Separate cohorts of mice were injected with tin protoporphyrin IX [SnPP, a heme oxygenase-1 (HO-1) inhibitor, 25 mg/kg, Tocris Bioscience] or vehicle (DMSO, 2.5 mL/kg, Sigma-Aldrich) 30 min before the oxypurinol first treatment (24). Hemin was dissolved in 0.1 M NaOH, titrated to pH 7.3 with 3.6% HCl, and diluted 1:8 with saline. Body temperature was maintained at 36.5–37°C using a surgical heating pad (FHC, Bowdoin, ME). Mice were euthanized 24 h after renal I/R injury with an overdose (200 mg/kg) of pentobarbital sodium. Kidney tissues were harvested 24 h post-operatively, and blood samples were taken from the vena cava.

### Measurement of kidney functional and histological damages

Twenty-four hours after surgery, we measured plasma creatinine (PCr) and blood urea nitrogen (BUN) levels using creatinine and urea nitrogen reagent kits (BioAssay Systems, Hayward, CA). To assess kidney histological damage, kidney hematoxylin-and-eosin (H&E)-stained sections after renal I/R or sham surgeries were evaluated by a pathologist who was blinded. The kidneys were analyzed using the following previously reported scoring method (25): 0, no damage; 1, mild damage with rounding of epithelial cells and dilated tubular lumen; 2, moderate damage with flattened epithelial cells, dilated lumen, and congestion of the lumen; and 3, severe damage with flat epithelial cells lacking nuclear staining and luminal congestion.

### Terminal deoxynucleotidyl transferase dUTP nick end labeling (TUNEL) assay and immunohistochemistry (IHC) staining

Renal tubular apoptosis was detected by TUNEL staining using a DeadEnd Fluorometric TUNEL System Kit (Promega, Madison, WI) according to the manufacturer's protocol. TUNEL-positive cells were counted in 5–8 randomly chosen 200 × microscopic fields. IHC staining was performed to confirm neutrophil infiltration and the generation of 8-hydroxy-2'-deoxyguanosine (8-OHdG), an oxidized nucleoside of DNA. The primary antibodies used were lymphocyte antigen 6 complex locus G6D (Ly6G, 1:100, eBioscience, San Diego, CA), cleaved caspase-3 (1:400, Cell Signaling Technology, MA) and 8-OHdG (1:500, Abcam, Cambridge, UK). The respective HRP-labeled secondary antibodies (BETHYL-Laboratories, Montgomery, TX) were used. Ly6G and cleaved caspase-3 positive cells were counted in 5–8 randomly chosen microscopic fields. The 8-OHdG densities were measured in 5–8 randomly chosen microscopic fields using Fiji Image J2 (NIH, Bethesda, MD), as described by Ruifrok et al. (26).

### Western blotting

Kidney samples were homogenized with a RIPA lysis buffer (50 mM Tris-HCl [pH 8.0], 1% Triton-X 100, 0.5% sodium deoxycholate, 0.1% SDS, 1 M NaF) plus protease inhibitor cocktail (Sigma-Aldrich, St. Louis, MO) and phosphatase inhibitor cocktail (Sigma-Aldrich). Protein samples were separated by SDS-PAGE and transferred to polyvinylidene difluoride (PVDF) membranes (GVS, Bologna, Italy). After blocking with 5% bovine serum albumin for 30 min, the membranes were incubated with antibodies against Ly6G (1:2,000, Fisher Scientific, Hampton, NH), 4-hydroxynonenal (4-HNE, 1:2000, Abcam), HO-1 (1:2,000, Cell Signaling Technology), and GAPDH (1:10,000, Bioworld Technology, St. Louis Park, MN) overnight at 4°C. The membranes were then incubated with their respective HRP-labeled secondary antibodies (1:3,000, BETHYL-Laboratories) for 1 h at room temperature. Protein expression levels were normalized to GAPDH. The protein band densities were then analyzed using ImageJ (NIH, Bethesda, MD).

### Quantitative RT-PCR

We measured HO-1 and catalase mRNA expression levels by quantitative RT-PCR. Total RNA was extracted from the kidney tissues using TRIzol reagent (Ambion, Austin, TX). The extracted RNA from each sample was synthesized as cDNA with random primers using reverse transcription PCR. cDNA levels were measured by quantitative RT-PCR (Biorad, Hercules, CA) using FastStart Universal SYBR Green Master Mix (Sigma-Aldrich), catalase-specific primers (sense primer 5'-GGTACACGCAAAAGGAGCA-3' and anti-sense primer 5'- TCCCACAAGATCCCAGTTACC-3'), and macrophage inflammatory protein (MIP)-2-specific primers (sense primer 5'-CCAAGGGTTGACTTCAAGAAC-3' and anti-sense primer 5'-AGCGAGGCACATCAGGTACG-3'). To check for equal RNA input, mRNA expression levels were normalized to GAPDH (sense primer 5'-ACCACAGTCCATGCCATCAC-3' and anti-sense primer 5'-CACCACCCTGTTGCTGTAGCC-3'). Relative mRNA expression was calculated using the ΔΔCt method. The specificity of the amplification was confirmed by melting curve analysis.

### Statistical analysis

Results were expressed as means ± standard errors of the mean (SEM). Data were analyzed using one-way ANOVA plus Tukey's *post-hoc* multiple comparison test and Student's *t*-test. The Mann–Whitney U test was used to analyze renal injury scores. Statistical significance was set at *P* < 0.05.

## Results

### Oxypurinol pretreatment protects the kidney against I/R injury *via* HO-1 induction

**Figure 1:**
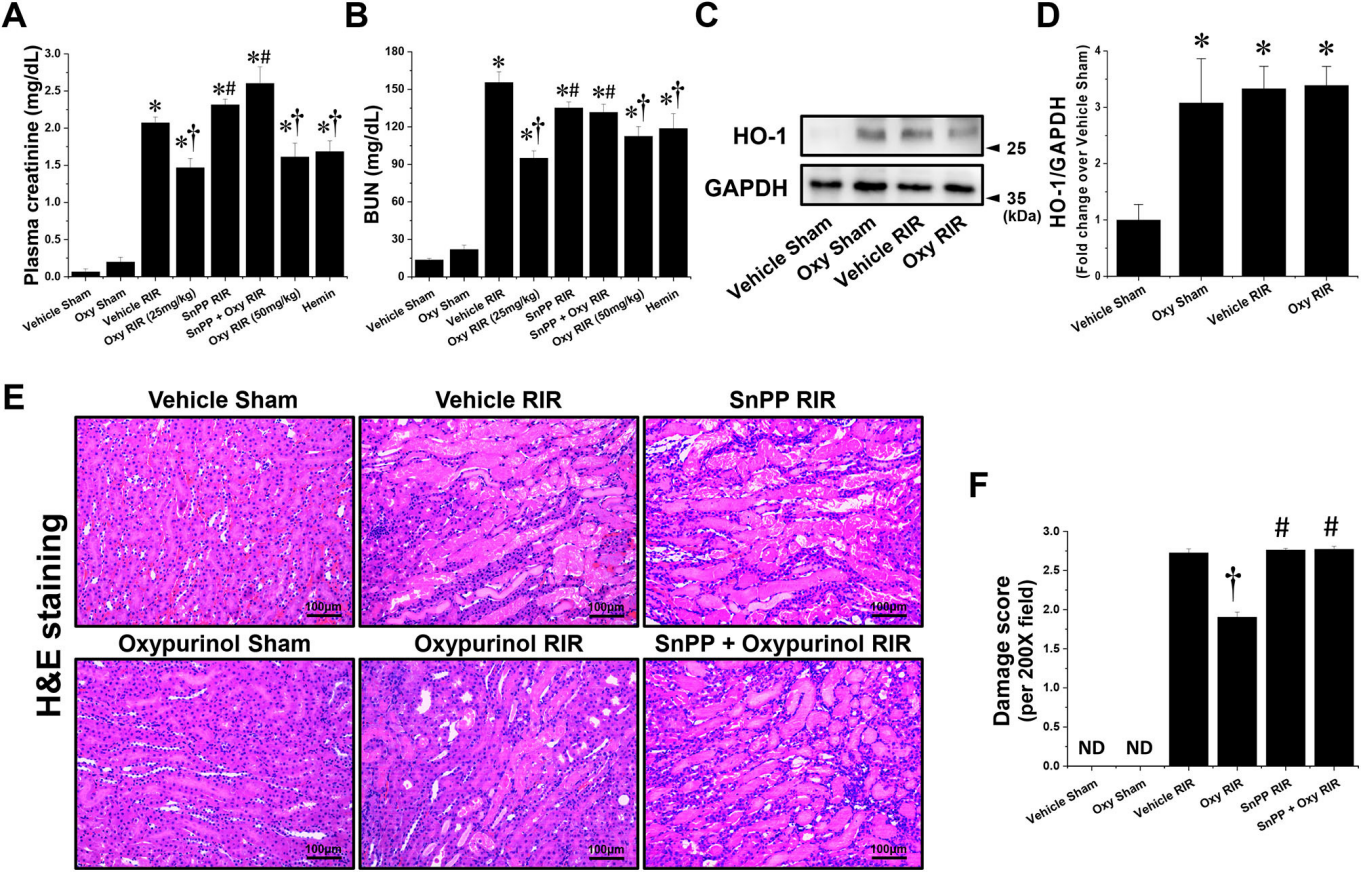
Oxypurinol protects against renal ischemia/reperfusion injury *via* heme oxygenase-1 (HO-1) induction. C57BL/6 male mice were subjected to sham or 30 min of renal ischemia surgeries. Some mice were pretreated with 25 or 50 mg/kg of oxypurinol at 24 h and 1 h preoperatively. To test whether HO-1 induction is critical for oxypurinol-mediated renal protection against renal I/R injury, we injected an HO-1 inhibitor [25 mg/kg, tin protoporphyrin IX (SnPP)] before oxypurinol first treatment. Some mice were pretreated with 25 mg/kg of hemin at 24 hours preoperatively. **(A, B)** Twenty-four hours after surgeries, plasma creatinine (PCr) and blood urea nitrogen (BUN) levels were measured. **(C)** Kidney samples were subjected to Western blotting using an anti-HO-1 antibody. GAPDH was used as a loading control. **(D)** Band intensities were measured using ImageJ. **(E)** Representative images (magnification, 200×) of kidney sections subjected to hematoxylin and eosin (H&E) are shown. **(F)** Histological damages were analyzed as described in the Materials and Methods section. Results are expressed as means ± standard errors of the mean (SEM) (vehicle or oxypurinol [oxy] sham *n* = 4; vehicle, oxypurinol, SnPP, or SnPP + oxypurinol renal ischemia/reperfusion (RIR) *n* = 6). *P < 0.05 vs. vehicle sham; ^†^*P* < 0.05 vs. vehicle RIR; ^#^*P* < 0.05 vs. oxypurinol RIR.

First, we assessed whether oxypurinol treatment protects against ischemic AKI in mice. Plasma creatinine (PCr) and blood urea nitrogen (BUN) levels were similar between vehicle- and oxypurinol-treated mice subjected to the sham operation (Figures 1A, B). As expected, PCr and BUN levels increased 24 h after renal I/R injury in the vehicle-treated mice. However, both 25 and 50 mg/kg oxypurinol or 25 mg/kg hemin-treated mice were significantly protected against kidney injury, as indicated by lower PCr and BUN levels. Since HO-1 plays a critical protective role in ischemic AKI by modulating kidney responses to injury (27) and since we found that oxypurinol significantly induced HO-1 protein expression in the kidneys (Figures 1C, D), we investigated whether oxypurinol protects the kidney against ischemic AKI *via* HO-1 induction. However, we couldn't find significant difference between vehicle RIR and oxypurinol RIR group. For this, we injected mice with tin protoporphyrin IX (SnPP), a selective HO-1 inhibitor, before oxypurinol treatment and found that pretreatment with SnPP significantly attenuated the protective effect of oxypurinol on renal I/R injury, as evaluated by PCr and BUN levels (Figures 1A, B).

Next, we assessed whether oxypurinol treatment protected kidney tubular cells from death after renal I/R injury. Vehicle-treated mice subjected to renal I/R showed severe loss of tubular nuclei (necrosis), and increased tubular congestion and dilatation. In contrast, oxypurinol treatment decreased renal tubular necrosis, congestion, and dilatation compared with vehicle treatment after renal I/R injury. However, pretreatment with SnPP before oxypurinol administration significantly offset the oxypurinol-mediated protective effect against histological renal tubular damage after I/R injury (Figures 1E, F).

### Oxypurinol pretreatment protects against apoptotic tubular cell death after renal I/R injury

**Figure 2:**
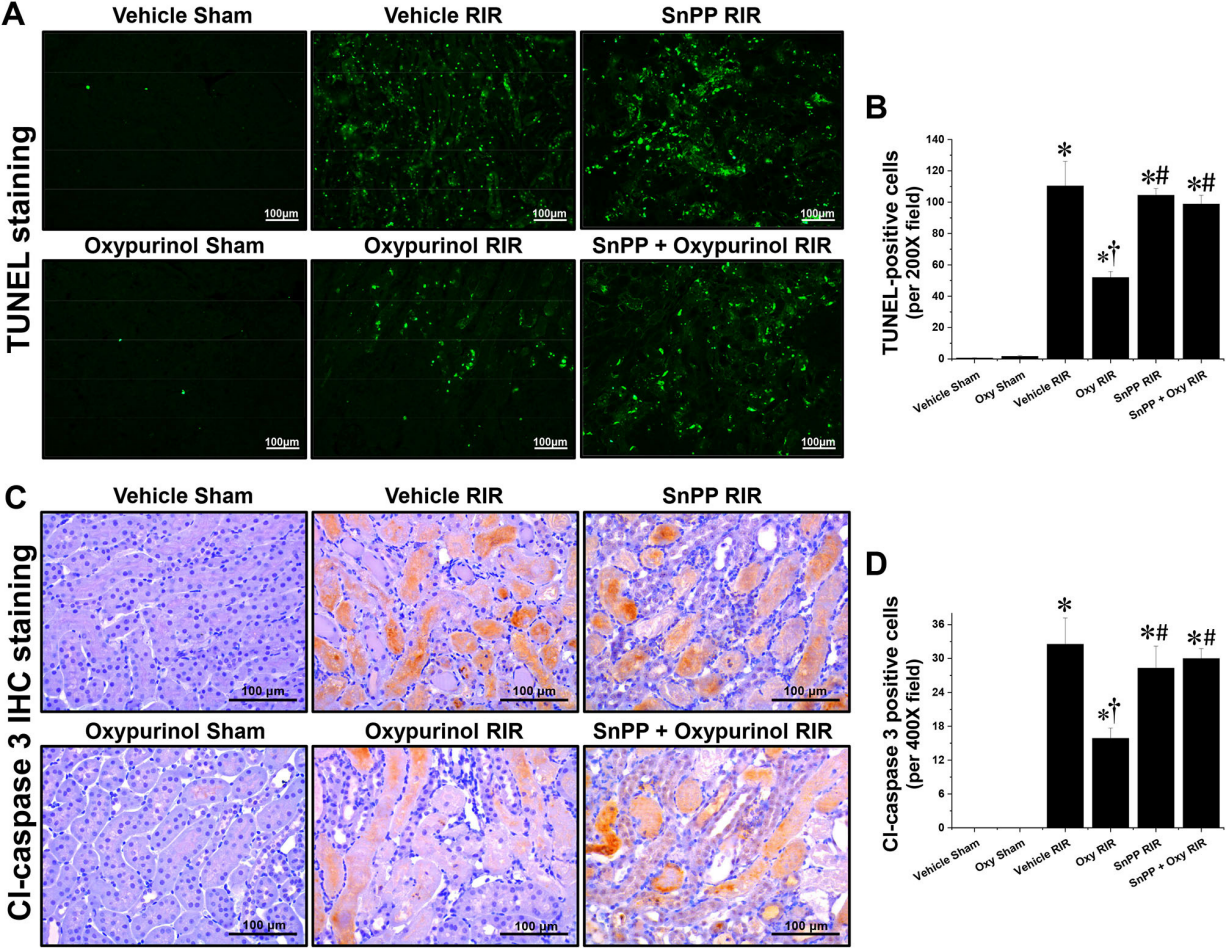
Oxypurinol protects renal tubular apoptotic cell death after renal ischemia/reperfusion injury. C57BL/6 male mice were subjected to sham or 30 min of renal ischemia surgeries. Some mice were pretreated with 25 mg/kg of oxypurinol 24 h and 1 h before surgeries. Separate cohorts of mice were injected with tin protoporphyrin IX [SnPP, a heme oxygenase-1 (HO-1) inhibitor, 25 mg/kg, Tocris Bioscience] 30 min before oxypurinol first treatment. **(A)** Representative images (magnification, 200×) of post-operative TUNEL staining in the kidneys are shown, and **(B)** the TUNEL-positive cells were counted. **(C)** Representative images (magnification, 400×) of immunohistochemistry staining using anti-cleaved-caspase 3 (Cl-caspase 3) antibody, and **(D)** the Cl-caspase 3-positive cells were counted. Results are expressed as means ± SEM (vehicle or oxypurinol [oxy] sham n = 4; vehicle, oxypurinol, SnPP, or SnPP + oxypurinol RIR *n* = 6). *P < 0.05 vs. vehicle sham; ^†^*P* < 0.05 vs. vehicle RIR; ^#^*P* < 0.05 vs. oxypurinol RIR.

Next, we evaluated apoptotic cell death, which is another major tubular cell death mechanism by TUNEL assay (Figures 2A, B), which is a method for detecting DNA fragmentation (28) and by immunohistochemistry staining (Figures 2C, D) using the antibody against cleaved caspase-3 which is a reliable marker for apoptosis as well as the final enzymatic cascade of apoptosis. Vehicle-treated mice subjected to renal I/R showed severe renal tubular apoptosis; however, oxypurinol treatment decreased this. SnPP pretreatment significantly prevented the oxypurinol-mediated protective effects against apoptosis after renal I/R injury.

### Oxypurinol pretreatment attenuates kidney neutrophil infiltration after renal I/R injury

**Figure 3:**
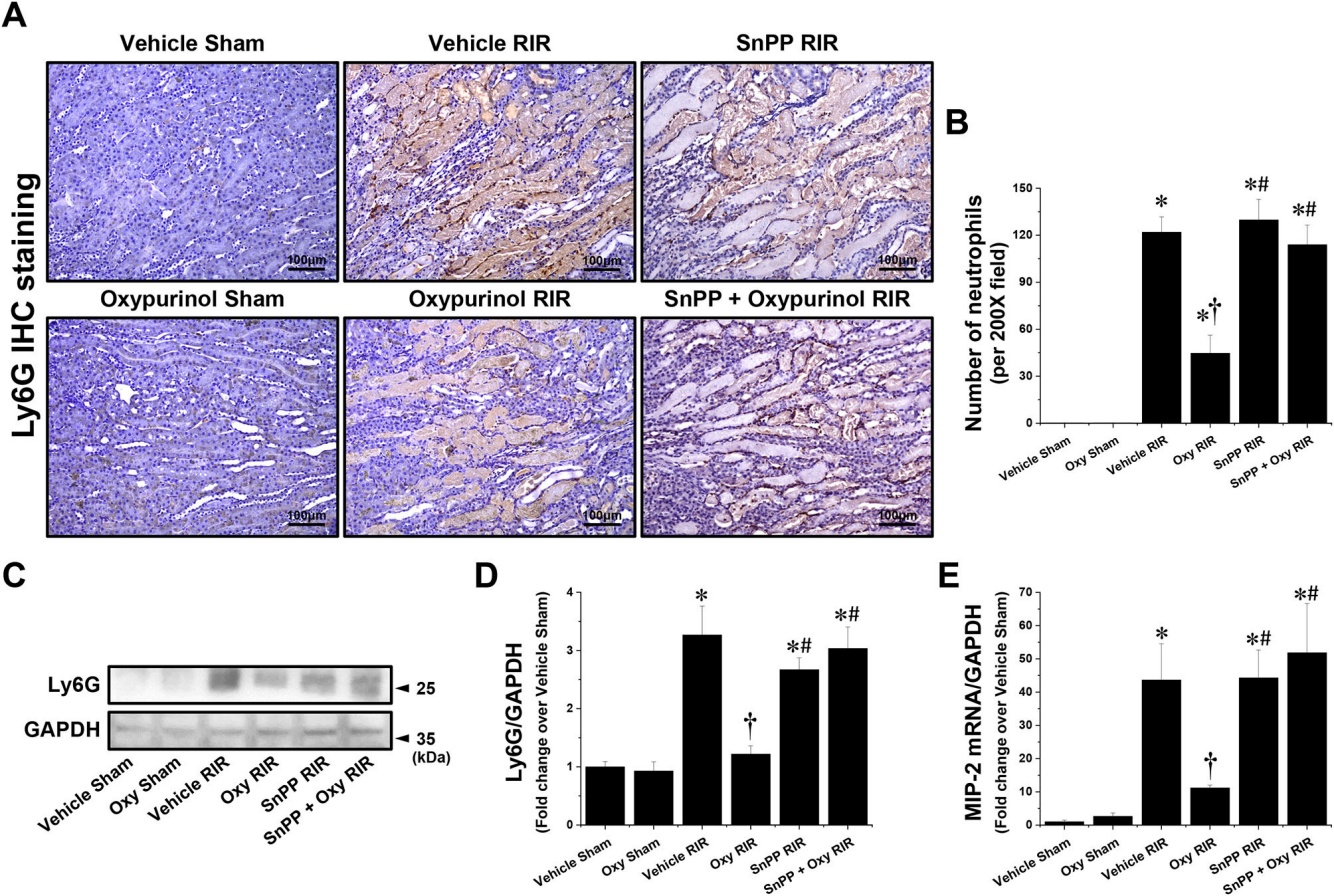
Oxypurinol reduces neutrophil infiltration and Ly6G protein expression after renal ischemia/reperfusion injury. C57BL/6 male mice were subjected to sham or 30 min of renal ischemia surgeries. Some mice were pretreated with 25 mg/kg of oxypurinol 24 h and 1 h before surgeries. Separate cohorts of mice were injected with tin protoporphyrin IX [SnPP, a heme oxygenase-1 (HO-1) inhibitor, 25 mg/kg, Tocris Bioscience] 30 min before oxypurinol first treatment. **(A)** Representative images (magnification, 200×) of immunohistochemistry staining in the post-operative kidneys show infiltrated neutrophils (dark brown), and **(B)** the Ly6G-positive cells were counted. **(C)** Kidney samples were subjected to Western blotting using an anti-Ly6G antibody. GAPDH was used as a loading control. **(D)** Band intensities were measured using ImageJ. **(E)** With quantitative RT-PCR, we measured the mRNA expression of MIP-2, which was normalized to GAPDH mRNA expression. Results are expressed as means ± SEM (vehicle or oxypurinol [oxy] sham *n* = 4; vehicle, oxypurinol, SnPP, or SnPP + oxypurinol RIR *n* = 6). *P < 0.05 vs. vehicle sham; ^†^*P* < 0.05 vs. vehicle RIR; ^#^*P* < 0.05 vs. oxypurinol RIR.

Next, we assessed whether oxypurinol treatment protects neutrophil infiltration by immunohistochemistry staining using the Ly6G antibody. Vehicle-treated mice subjected to renal I/R showed markedly increased neutrophil infiltration near the outer stripe of the outer medulla, and was decreased by oxypurinol treatment (Figures 3A, B). Similarly, the protein expression of Ly6G (Figures 3C, D), as evaluated by Western blotting, and the mRNA expression of MIP-2 (Figure 3E), as evaluated by RT-PCR, increased in renal I/R injury; however, oxypurinol pretreatment reduced the protein expression of Ly6G and the mRNA expression of MIP-2 after renal I/R injury. However, pretreatment with SnPP before oxypurinol administration significantly offset the oxypurinol-mediated protective effect against upregulation of Ly6G protein and MIP-2 mRNA expressions after I/R injury (Figure 3).

### Oxypurinol pretreatment attenuates oxidative damage after renal I/R injury

**Figure 4:**
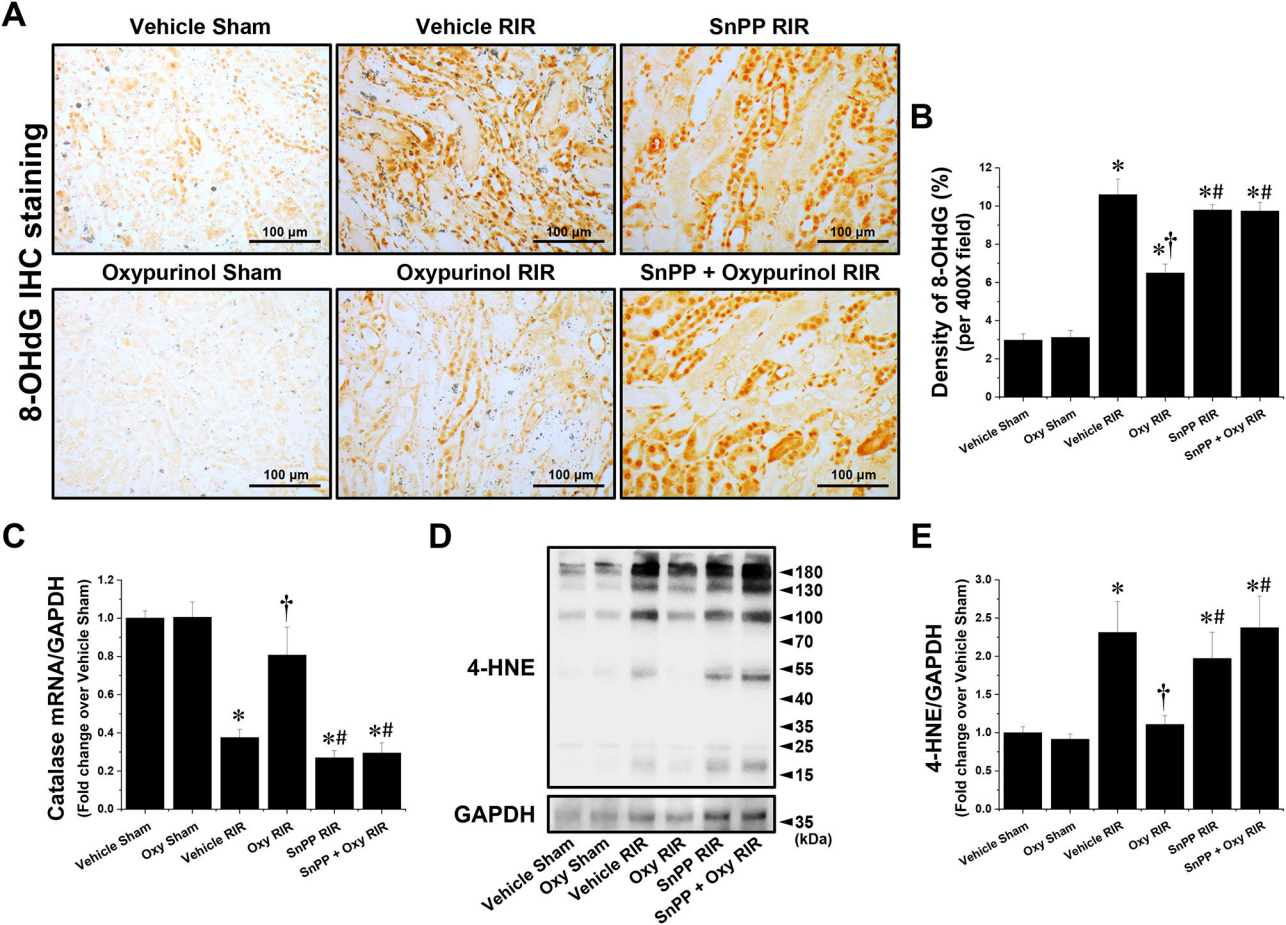
Oxypurinol reduces oxidative damage after renal ischemia/reperfusion injury. C57BL/6 male mice were subjected to sham or 30 min of renal ischemia surgeries. Some mice were pretreated with 25 mg/kg of oxypurinol 24 h and 1 h preoperatively. Separate cohorts of mice were injected with tin protoporphyrin IX [SnPP, a heme oxygenase-1 (HO-1) inhibitor, 25 mg/kg, Tocris Bioscience] 30 min before oxypurinol treatment. **(A)** Representative images (magnification, 400×) of 8-OHdG (dark brown) show immunohistochemistry staining in the post-operative kidneys. **(B)** Densities of 8-OHdG staining were measured using the Fiji Image J2 software as described. **(C)** With quantitative RT-PCR, we measured the mRNA expression of catalase, an antioxidant enzyme. Catalase mRNA expression was normalized to GAPDH mRNA expression. **(D)** Kidney samples were subjected to Western blotting using an anti-4-Hydroxynonenal (4-HNE) antibody. GAPDH was used as a loading control. **(E)** Band intensities were measured using the ImageJ software. Results are expressed as means ± SEM (vehicle or oxypurinol [oxy] sham *n* = 4; vehicle, oxypurinol, SnPP, or SnPP + oxypurinol RIR *n* = 6). *P < 0.05 vs. vehicle sham; ^†^*P* < 0.05 vs. vehicle RIR; ^#^*P* < 0.05 vs. oxypurinol RIR.

Figures 4A, B show representative immunohistochemistry images for 8-OHdG, a marker of oxidative stress to DNA, and the density of 8-OHdG in the kidneys of each group of mice. Vehicle-treated mice subjected to renal I/R had a markedly increased density of 8-OHdG near the outer stripe of the outer medulla, but oxypurinol treatment decreased the density of 8-OHdG after renal I/R injury (Figures 4A, B). In contrast, SnPP pretreatment significantly prevented the oxypurinol-mediated protective effects against oxidative DNA damage after renal I/R injury (Figures 4A, B). Furthermore, we evaluated the fold-change in the mRNA expression of catalase, an antioxidant enzyme, using quantitative RT-PCR. Catalase mRNA expression decreased after renal I/R injury, but oxypurinol treatment prevented the decrease in mRNA expression of catalase in the kidneys (Figure 4C). In contrast, SnPP pretreatment significantly prevented the oxypurinol-mediated protective effects against mRNA expression of catalase after renal I/R injury. Additionally, Figures 4D, E show that the expression of 4-HNE which is an indicator of lipid peroxidation and 4-HNE modification occurs at several amino acids side chains in a variety of proteins during oxidative stress (29), was significantly increased in the I/R-injured kidneys, and oxypurinol-treated mice subjected to I/R had decreased lipid peroxidation. In contrast, SnPP pretreatment significantly prevented the oxypurinol-mediated protective effects against lipid peroxidation after renal I/R injury.

## Discussion

Oxidative stress is one major pathogenic mechanism of AKI, and occurs when the level of reactive oxygen species (ROS) exceeds that of defensive antioxidant systems. ROS produced by several sources, including mitochondria, xanthine oxidase, and NADPH oxidase (30) causes renal dysfunction, tubular necrosis, and apoptosis (31, 32). Oxidative stress biomarkers include 4-hydroxynonenal (4-HNE) and 8-hydroxy-2'-deoxyguanosine (8-OHdG). 4-HNE is an important marker of lipid peroxidation that is produced under oxidative stress (33). Additionally, 8-OHdG is a marker of oxidative DNA damage, including nucleic and mitochondrial DNA (34). Oxypurinol has a potential antioxidant effect to remove both hydroxyl radicals and hypochlorous acid (35, 36). Indeed, oxypurinol is well-known an inhibitor of xanthine oxidase which generate ROS such as hydrogen peroxide and superoxide during oxidation of xanthine, hypoxanthine and other purines (36). Because one of the principal mechanisms of renal I/R injury is an excessive production of ROS, and the protective effect of xanthine oxidase inhibitors such as febuxostat and allopurinol on ischemic AKI have been reported (11, 37), it is possible that oxypurinol protects ischemic AKI *via* xanthine oxidase inhibition.

Together with oxidative stress, inflammation is also a critical pathogenic mechanism of ischemic AKI, and oxidative stress and inflammation are tightly interrelated during AKI development because ROS-induced oxidative damage recruits inflammatory cells, such as neutrophils and macrophages, leading to additional renal damage, cell death, and dysfunction (38). In contrast, anti-inflammatory drugs also reduce oxidative stress because leukocytes induce kidney injury by releasing ROS, myeloperoxidase, proteinases, elastases, and cationic peptides, which can induce oxidative damage directly or indirectly (39). Among the inflammatory cells, neutrophils are the earliest to accumulate in the kidney and are crucial mediators in the development of ischemic AKI (39). In several AKI models, including I/R and cisplatin, neutrophil accumulation is reduced by anti-inflammatory drugs, and prevention of neutrophil tracking to the kidney lowers renal damage (40, 41). Indeed, blocking neutrophils alleviated the severity and duration of AKI. In this study, we found that oxypurinol significantly decreased 8-OHdG, 4-HNE expression and catalase mRNA expression, as well as tubular cell apoptosis and necrosis in renal I/R models. Furthermore, oxypurinol-treated mice showed significantly decreased neutrophil infiltration, protein expression of Ly6G, a neutrophil marker, and mRNA expression of MIP-2, also known as CXC ligand (CXCL)2 which recruits polymorphonuclear neutrophils which are the earliest to accumulate in the kidney and are crucial mediators in the development of ischemic AKI (39) after renal I/R compared with vehicle-treated mice. These results demonstrated that oxypurinol treatment has a protective effect against kidney damage by reducing the oxidative stress, inflammation, and cell death during renal I/R injury.

HO-1 is a major cytoprotective enzyme that generates oxidative cleavage of heme groups, leading to carbon monoxide, biliverdin, and iron (42). HO-1 has received significant attention in treating numerous human diseases, including AKI. HO-1 exerts protective effects in AKI animal models induced by renal I/R (43, 44), ureteral obstruction (45), cisplatin (46, 47), and LPS (48). For example, Chen et al. demonstrated that HO-1 activation by hemin pretreatment prevents renal I/R injury through ERK 1/2-enhanced tubular epithelium proliferation (8). Rossi et al. (49) and Correa-Costa (50) also demonstrated that pretreatment of hemin mitigates renal I/R injury induced acute kidney injury. Consistent with those previous studies, we confirmed that the HO-1 activation by hemin pretreatment protected ischemic kidney injury (Figures 1A, B). *The hmox1* gene encoding HO-1 is regulated by several transcription factors, including heat shock factor (HSF), NF-κB, nuclear factor erythroid 2-related factor 2 (Nrf2) and activator protein−1 (AP-1) families, and mitogen-activated protein kinases (p38, ERK, and JNK) (51). Of these *hmox1* transcription factors, oxypurinol was reported to induce HO-1 *via* p38 phosphorylation in cultured THP-1 cells (52), but we failed to detect p38 phosphorylation by oxypurinol treatment in both our *in vivo* and *in vitro* experiment settings (data not shown). Allopurinol, a precursor of oxypurinol, activates Nrf2, a major transcription factor of antioxidants, including HO-1, NAD(P)H quinone oxidoreductase 1, and glutathione S-transferase. In this study, we found that oxypurinol significantly induced HO-1 protein expression in the sham kidneys, but we couldn't find significant difference between vehicle RIR and oxypurinol RIR groups. Because we and other researchers confirmed that renal I/R injury itself can induce HO-1 expression as a protective mechanism (8, 53), we speculate that the sum of increase in HO-1 expression by mild renal IR injury and HO-1 induction by oxypurinol would be similar to the increase in HO-1 expression by severe renal I/R injury. However, we demonstrate that direct HO-1 suppression by tin protoporphyrin IX administration entirely eliminated the oxypurinol-mediated protection against ischemic AKI, suggesting that the renal protective effects of oxypurinol are at least in part mediated by HO-1 induction.

Our renal I/R model is a leading cause of perioperative AKI in various clinical settings such as major vascular, cardiac and hepatic surgeries, and kidney transplantation (2). So, it is possible to adapt kidneys to renal I/R injury before those clinical surgeries by preconditioning such as short ischemia, remote organ ischemia, and treatment of pharmacological drug including HO-1 activators. Therefore, our findings suggest that oxypurinol-mediated preconditioning *via* HO-1 induction protects ischemic AKI rather than recovers from ischemic AKI by attenuating necrosis, apoptosis, inflammation, and oxidative damage after I/R, suggesting that oxypurinol and its underlying mechanism may be potential preventive drug for ischemic AKI.

## Data availability statement

The raw data supporting the conclusions of this article will be made available by the authors, without undue reservation.

## Ethics statement

The animal study was reviewed and approved by Institutional Animal Care and Use Committee (IACUC) of Pukyong National University.

## Author contributions

HK and SH conceived and designed research, prepared figures, and drafted manuscript. HK performed experiments. HK, CL, JK, and SH analyzed data, interpreted the results of experiments, edited and revised manuscript, and approved final version of manuscript. CL and JK provided material. All authors contributed to the article and approved the submitted version.
